# Grouping-Induced Numerosity Biases Vary with Autistic-Like Personality Traits

**DOI:** 10.1007/s10803-021-05029-1

**Published:** 2021-04-28

**Authors:** Antonella Pomè, Camilla Caponi, David Charles Burr

**Affiliations:** 1 Department of Neuroscience, Psychology, Pharmacology, and Child Health, University of Florence, Padiglione 26, Via di San Salvi, 26, 50135 Florence, Italy; 2 School of Psychology, University of Sydney, Sydney, NSW Australia

**Keywords:** Number perception, Segmentation, Perceptual grouping, AQ

## Abstract

Individuals with autism spectrum disorder are thought to have a more local than global perceptual style. We used a novel paradigm to investigate how grouping-induced response biases in numerosity judgments depend on autistic-like personality traits in neurotypical adults. Participants judged the numerosity of clouds of dot-pairs connected by thin lines, known to cause underestimation of numerosity. The underestimation bias correlated strongly with autism-spectrum quotient (r = 0.72, Bayes factor > 100), being weaker for participants with high autistic traits. As connecting dots probably activates global grouping mechanisms, causing dot-pairs to be processed as an integrated whole rather than as individual dots, the results suggest that these grouping mechanisms may be weaker in individuals self-reporting high levels of autistic-like traits.

## Introduction

Over the last few decades, a large body of research has addressed the issue of visual processing of scenes at global or local levels of structure (Wagemans et al., 2012, for review). In particular, individuals with autism spectrum disorder (ASD) often show a perceptual style privileging local detail over global integration. These reports show superior performance in autistic children and adults on tasks where fine-grained visual features must be abstracted from their global context, such as the embedded figure task (Shah & Frith, 1983) or the Navon figures (Plaisted et al., 1999). Studies also suggest that within the typically developing population, people with high autistic-like tendencies, as defined by the autism-spectrum quotient (AQ) (Baron-Cohen et al., 2001), show a more local perceptual style. For example, Turi et al. (2018) reported that participants with high AQ perceived a bistable rotating cylinder in a more local way than those with low AQ. Pupil diameter for high AQ participants modulated in phase with the bistable perception of the bistable cylinder, dilating when the black surface was in front and constricted when white was in front, consistent with a local detail-oriented perceptual style where the front surface dominated perception. Those with low AQ showed much less phase-locked pupil modulation, consistent with a more global perceptual style.

The local processing visual style has led to theories of autism such as *enhanced perceptual functioning* (Mottron et al., 2006) and *weak central coherence* (Happé & Frith, 2006). Both highlight local bias in visual processing, and this bias which can lead to superior performance on some tasks (O’Riordan & Plaisted, 2001; Shah & Frith, 1993). Weak central coherence further assumes that the local bias is accompanied by a deficit in processing global configuration in autism, potentially affecting important visual skills that require the representation of multiple elements.

Pellicano and Burr (2012) attempt to explain the preference for local structure within a more general *Bayesian* framework, which assumes that perception depends not only on current input but is also shaped by perceptual experience (*priors*) and other contextual information*.* They suggested that autistic perception is dominated more by direct sensory information than past experience and other contextual cues, and therefore in some sense “more real” than neurotypical perception. Past experience could provide useful models to guide global perception, explaining, at least in part, the local preference with autism. Incorporating spatial contextual cues could also lead to a more global take on the perceptual scene.

Although it is generally assumed that perception is less global in autism, there is little direct evidence for this assertion. One important issue for global perception is segmentation and grouping of images into objects. Visual grouping has been a significant focus of perception research since it was first emphasized by Gestalt psychologists (Arnheim & Metzger, 1977; Wertheimer, 1938) who proposed several simple grouping rules that may influence perception, including *proximity*, *similarity*, *good continuation*, and *connectedness*. These Gestalt principles cause the grouped objects to appear to ‘belong together’ and be processed as a whole and facilitating perception.

Grouping affects many aspects of global perception, including the perception of numerosity: when visual items—such as circles or squares—are grouped together with connecting lines, they appear less numerous (Franconeri et al., 2009; He et al., 2015), evidence that numerosity operates on segmented objects, rather than individual local elements. The connections between elements can be quite weak: thin lines connecting two dots causes the same reduction in apparent numerosity as do solid connections creating realistic 3D shapes (Franconeri et al., 2009). For densely packed items, the effect of connectivity is greatly reduced (Anobile et al., 2017), showing that the effect is limited to the range of estimation of numerosity, not texture density. The illusion has been extensively studied in neurotypical adults, with both psychophysical and neurophysiological techniques, which have shown that connecting dots changes not only the apparent numerosity of a dot-cloud, but also the neural representation of number (He et al., 2009). It also affects adaptation to number (Fornaciai et al., 2016), and pupillometry (Castaldi et al., 2021). All studies show that the connectedness bias does not result from response biases or cognitive artifacts but reflects real neuronal changes: and these neuronal changes covary strongly with autistic-like personality traits.

Given the lack of direct evidence for weakened global perception in autism, we used this numerosity illusion to measure perceptual grouping. The illusion taps grouping mechanisms indirectly, without having to ask participants to report perceptual organization. We measured the apparent numerosity of clouds of dots connected with thin lines to form dumbbell-like shapes, in a group of young neurotypical adults with varying autistic-like traits. We correlated the magnitude of the effect against AQ-defined autistic traits, reasoning that those with higher autistic traits would be less sensitive to the global grouping information of the connecting elements. Therefore, they should show less biased discrimination of numerosity compared to those with lower autistic traits, who should show more substantial underestimation of the connected stimulus.

## Methods

### Participants

Twenty-one neurotypical young psychology students from the University of Florence, and all naïve to the goals of the experiment [11 females, age: 27.3 ± 2.8 (mean ± SD)]. All had normal or corrected-to-normal visual acuity without major visual impairment, and two were left-handed (but used their right hand for the experiment). All participants gave written informed consent, and experimental procedures were approved by the local ethics committee (*Comitato Etico Pediatrico Regionale—Azienda Ospedaliero-Universitaria Meyer*, Florence), and are in line with the declaration of Helsinki.

### AQ Scores

All participants completed the self-administered Autistic Quotient questionnaire, in either the validated Italian or English versions (Ruta et al., 2012; Ruzich et al., 2015). This contains 50 items, grouped in five subscales: attention switching, attention to detail, imagination, communication and social skills. For each question, participants read a statement and selected the degree to which the statement best described them: “strongly agree,” “slightly agree,” “slightly disagree,” and “strongly disagree”. The standard scoring described in the original paper was used: 1 when the participant’s response was characteristic of ASD (slightly or strongly), 0 otherwise. Total scores ranged between 0 and 50, with higher scores indicating higher degrees of autistic traits. All except two participants (with AQ 33 and 32) scored below 32, the threshold above which a clinical assessment is recommended (Baron-Cohen et al., 2001). The median of the scores was 15, with lower and upper quartiles of 11 and 26. Scores were normally distributed, as measured by the Jarque–Bera goodness-of-fit test of composite normality (JB = 1.75, p = 0.17).

### Stimuli and Procedure

Perceived numerosity was measured with a two alternative forced choice method (2AFC). Participants sat 57 cm from a calibrated 13.3-inch monitor (resolution 2560 × 1600 pixels; refresh rate 60 Hz), and reported by keypress just after stimulus disappearance which of two clouds of dots appeared to be more numerous, guessing when uncertain. Stimuli were presented simultaneously either side of a central fixation point for 500 ms, too fast for single elements to be serially countable. One of the two dot clouds was the *reference* (randomly left or right), which had fixed numerosity throughout the experimental session (15, 25, 50 or 100 dots); the other was the *probe*, which varied in numerosity, guided by the adaptive Quest routine (Watson & Pelli, 1983). After each trial, the QUEST routine estimated the point of subjective equality (PSE) which, after being perturbed by a random number drawn from a normal distribution of standard deviation 0.5 dots (to span a range around PSE), served as the probe numerosity of the next trial. This procedure automatically ensured equated the number of “greater than” and “less than” responses. Each participant performed 180 trials for each base numerosity, for both isolated and connected dots (see below), resulting in a total of 1440 trials for each participant. Stimuli were generated with Matlab 9.1 using PsychToolbox routines (Brainard, 1997).

**Fig. 1:**
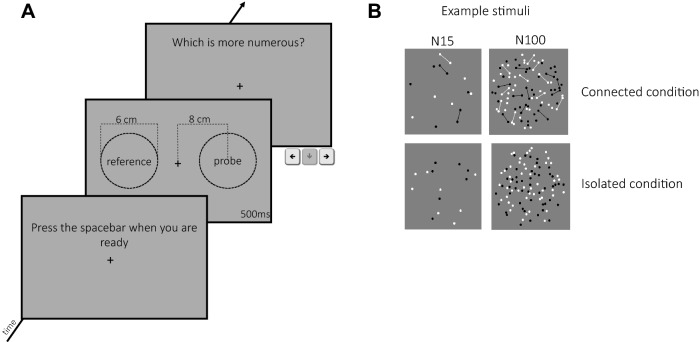
Paradigm and example stimuli used. **a** Observers viewed the screen from 57 cm, so 1 cm = 1 degree of visual angle. Each trial started with a central fixation cross. At will the observer initiated the trial by button-press, and two dot clouds were displayed together for 500 ms, straddling the fixation cross at the distances shown in the figure. After stimulus disappearance, they indicated which cloud of dots seemed more numerous, pressing the appropriate key on a keyboard. The response was unspeeded, but excessively long or short responses (more than 2 standard deviations from mean) were eliminated. **b** Example stimuli showing the connected condition (upper images), with 40% dots connected by thin lines, and for the isolated-dot condition (lower images). The left-hand images had low numerosity (N = 15), the right-hand images the highest numerosity (N = 100)

Dots were small disks of 2.5 mm diameter (subtending 0.25° at 57 cm), half-white, half-black (so that luminance did not vary with number, providing a potential cue). The experiment used two types of random dot-patterns, illustrated in Fig. 1b: dots were either *isolated* (lower images in Fig. 1b), or with 40% of neighboring dots *connected* to create dumbbell-like shapes (upper images). For patches containing isolated dots, dot positions were generated on-line to respect the sole condition that two items could not be closer than 2.5 mm (0.25°), preventing dot overlap. For the connected patterns, dot position was calculated in two stages: first couples of dots (40% of the total dots of the reference stimulus) were cast and connected via a line of the same color, with the constraints that line length was between 10 and 15 mm, with no lines crossing; in the second stage, the remaining 60% of dots were cast with the constraint of not overlapping either the other dots or the connecting lines. The connector line width was 0.5 mm. The probe stimuli always comprised only *isolated* dots, but the constant-numerosity reference could comprise either isolated (baseline) or *connected* dots.

### Data Analysis

Data were analyzed separately for each subject. To avoid anticipatory or over-late responses, data with reaction times that exceeded two standard deviations above or below the mean time taken to give a response were removed (less than 2% of the trials).

For each condition (*connected* or *isolated* dot-clouds) and reference numerosity, the responses were plotted as function of the probe numerosity and fit with a cumulative Gaussian distribution, whose median defines the PSE and the difference in numerosity between 50 and 75% correct responses defines the JND, a measure of precision. The JND divided by the perceived numerosity yields the Weber fraction (WF), a dimensionless index of imprecision that allows comparison of performance across numerosities. As we were interested in the effect of autistic personality traits on the results, we divided participants into low AQ (displayed as red) and high AQ (blue), based on a median split of their AQ scores (above or below 15). Our main analyses compared data across conditions and groups of participants: standard t-tests, ANOVAs and correlation analyses were complemented with Bayes factors estimation. Bayesian analyses were performed with the software JASP (entered with the per-condition and per-subject averages computed in Matlab). Bayes Factors (Rouder et al., 2009) quantify the evidence for or against the null hypothesis as the ratio of the likelihoods for the experimental and the null hypothesis. We express it as the base10 logarithm of the ratio, where negative numbers indicate that the null hypothesis is likely to be true, positive that it is more likely false. By convention, absolute log_10_ Bayes factors > 0.5 are considered substantial evidence for either the alternate or null hypothesis, > 1 strong evidence, and > 2 decisive.

## Results

**Fig. 2:**
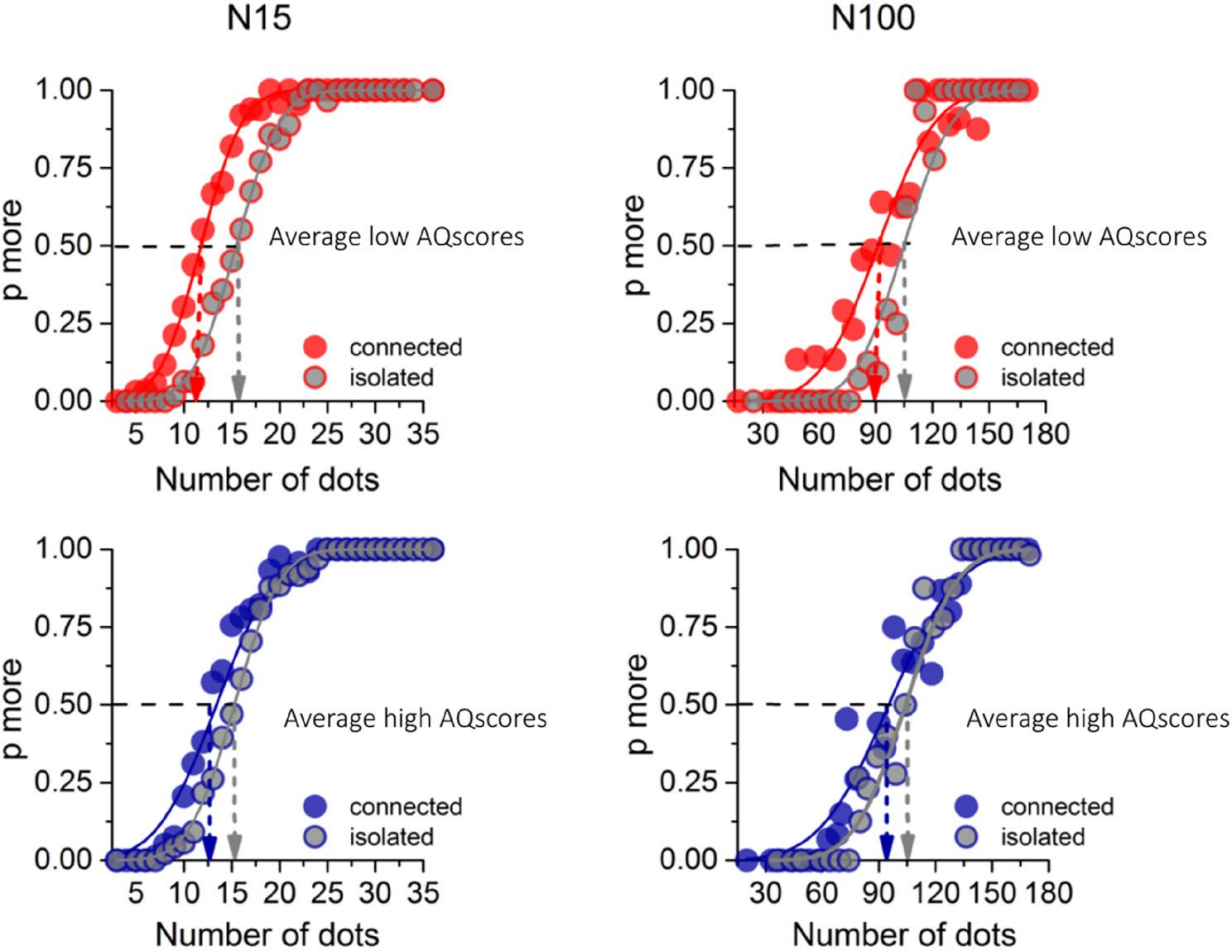
Aggregate psychometric functions for low and high autistic traits. Psychometric functions for reference N = 15 (left panels) and N = 100 (right panels) for the aggregate of participants with low AQ (< 15; N = 11) in red and the pooled high AQ group (N = 10) in blue. The functions plot the proportion of trials where the probe appeared more numerous than the reference (either 15 or 100 dots), as function of probe numerosity (shown in the abscissa). The vertical dashed lines show the estimates of the PSE, given by the median of the fitted cumulative Gaussian functions. Gray filled circles refer to baseline conditions, red and blue circles to connected conditions

As described above, participants were required to discriminate which of two simultaneously presented dot patterns—a reference and a probe—appeared to be more numerous. The variable probe was always a pattern of isolated dots, while the constant reference dots could be either isolated (baseline condition) or connected, in different blocks (see Fig. 1b). Figure 2 shows psychometric functions for numerosity discrimination for two numerosities (15 and 100), for isolated and connected dots, separately for low- and high-AQ participants. These functions plot *aggregate data* pooled over the two groups of participants to illustrate the effects and also the technique. However, all subsequent analyses were done on individual data after computing similar functions for each participant.

For the 15-dot stimuli, there is a clear effect of connecting dots, particularly for participants of low AQ (Fig. 2a). The point of subject equality (PSE) for the isolated dots was very near the physical numerosity of the reference (N15), as to be expected. However, when 40% reference dots were connected, the probe PSE was only 11 for the low-AQ group, 27% less than the physical numerosity, agreeing with previous literature (Anobile et al., 2017; Franconeri et al., 2009; He et al. 2009, 2015; Kirjakovski & Matsumoto, 2016). However, for the high AQ group (Fig. 2b), the shift was much reduced, only about 2 elements, or 13%. As was observed before (Anobile et al., 2017), the connectedness effect becomes much reduced at high numerosities. For 100-dot displays, the connectivity effect (difference between the isolated and connected PSEs) was 11% for the low-AQ group and 8% for the high.

**Fig. 3:**
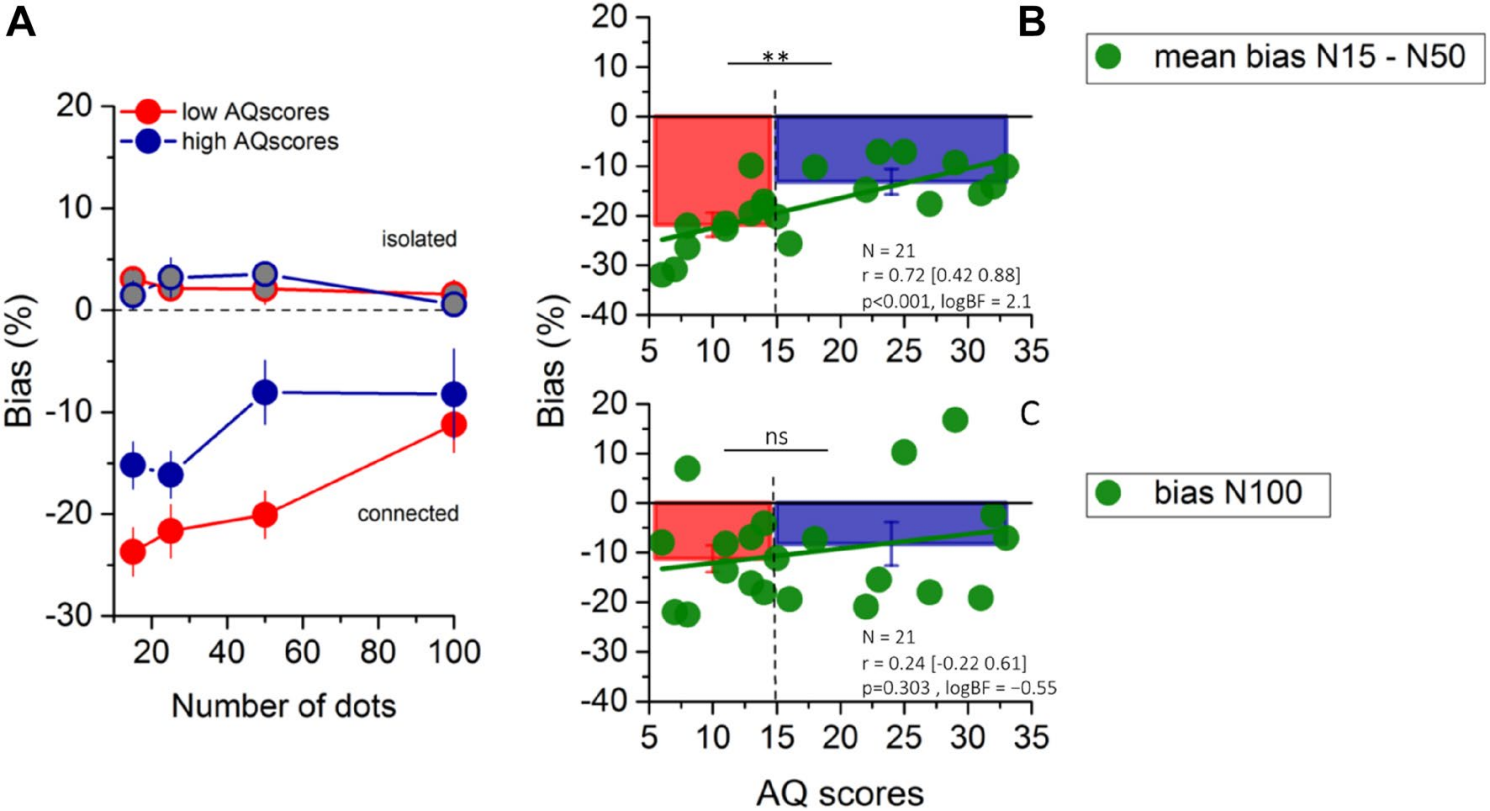
Response bias as a function of numerosity, and correlation with Autistic Quotient. **a** Average PSEs as a function of dot number, expressed as percentage difference from the reference number, color coded as in Fig. 2. **b** Mean bias at low numerosities (N15–N50) plotted against AQ for all participants. Bar graphs show the mean underestimation bias for low (in red) and high AQ (in blue), with error bars =  ± 1 SEM. Vertical dashed lines show the median split of the scores. Significance values refer to two-sample T-test (**p < 0.01, ns p > 0.5). Text insets report Pearson’s r values and associated p-values and Bayes Factors. Thick green lines show the linear fit through the data

The main analyses were performed on the data of individual participants. For each participant and condition, we fitted psychometric functions like those of Fig. 2, from which we extracted estimates of PSE for the various conditions. We then calculated the biases of the PSEs for the connected and baseline conditions. Figures 3a reports the average biases separately for the low and high AQ groups (color-coded as in Fig. 2). For both groups, the baseline bias (grey filled circles) was statistically indistinguishable from zero (p > 0.5), as to be expected. And for both groups, the bias of the connected stimuli decreased with numerosity. However, the magnitude of the illusion was much less reduced for the high- than the low-AQ subsample. This difference is revealed by the statistically significant interactions between numerosity and biases (two-way repeated measures ANOVA: F(3,57) = 7.91; p < 0.001; logBF = 31).

As has been previously reported, and is clear from the data of Fig. 3, connecting 40% of dots of the low-density patterns reduces apparent numerosity considerably, while at high densities the effect is less obvious for both groups. We therefore separated the data into low (N15–N50) and high (N100) numerosities to examine in more detail the relationship between biases and AQ. Figure 3b plots the mean bias for the connected patch at low numerosities against AQ scores, showing a very strong correlation, accounting for half the variance (r = 0.72; p < 0.001; logBF = 2.1): underestimation of the connected patches decreased with AQ scores, with Bayes factor consistent with “decisive” evidence. The difference is also seen in the mean underestimation effect for the low- and high-AQ groups shown in the overlaying bar graphs. The difference between the groups was significant, with Bayes factor pointing to strong evidence [t(19) = 3.32, p < 0.01, logBF = 1.08]. Figure 3c shows the bias at high densities (N100). Here there is no correlation with AQ [r = 0.24; p = 0.303; logBF =  − 0.55], and no significant difference between the average bias of the two groups [t(19) = 0.62, p = 0.54, logBF =  − 0.56]. For both measures Bayes factors show substantial evidence for no effect.

**Fig. 4:**
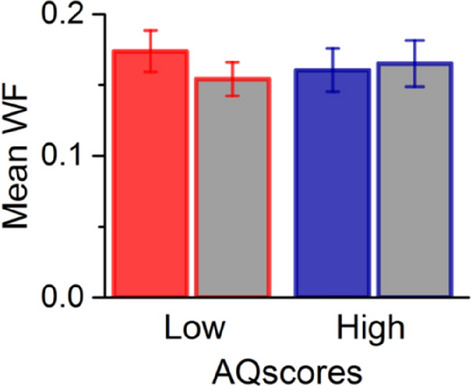
Mean Weber fraction for discriminating numerosity in the baseline (grey filled bars) and connected conditions for the two groups. Weber fractions are similar between low and high AQ groups. Error bars =  ± 1 SEM. There are no significant differences between conditions or groups

Figure 4 reports the WF of the participants (averaged over all numerosities), given by the SD of the best-fitting Gaussians to the psychometric functions, normalized by the average perceived quantity. The data show similar discrimination precision between the groups, with no difference between the two conditions tested. These results are confirmed by a two way ANOVA, which revealed no main effects of task [F(1,35) = 1.26; p = 0.27; logBF =  − 0.41] and AQ [F(1,35) = 0.75; p = 0.39; logBF =  − 0.64] nor a significant interaction between the two [F(1,35) = 1.3; p = 0.26; logBF =  − 1].

## Discussion

This study examined how perceptual grouping varies with autism traits, using a novel perceptual test: a numerosity illusion where connecting pairs of adjacent dots reduces the apparent numerosity of the dot-cloud, presumably by invoking grouping mechanisms that cause the connected dot-pairs to be grouped and perceived as a single unit. We showed that for low-moderate numerosities, connecting pairs of dots caused large changes in apparent numerosity, and the magnitude of the effect covaried strongly with AQ-defined autistic traits, with a correlation coefficient as high as 0.72, accounting for more than half the variance. The Bayes factor was > 100, “decisive evidence” in favor of the correlation. The results confirmed a previous study (Anobile et al., 2017) showing that the effect of connection occurs primarily at low-to-moderate numerosities, and importantly showed that the connectivity effect at moderate numerosities was far weaker in individuals scoring high on the AQ scale.

The generally accepted explanation for the connectedness effect is that number estimation operates over a segmented collection rather than at the level of individual dots or other simple features (Franconeri et al., 2009; He et al., 2015), a rapid and vastly parallel process, operating simultaneously over the entire extent of the display. The current results suggest that this segmentation process is weaker in individuals with strong autistic-like traits, reflecting a major difference between autistic and typical numerosity perception. At higher densities, the objects are presumably too “crowded” to be efficiently segregated (by either group of participants), and other processes come into play. Indeed there is considerable evidence that at these higher densities numerosity judgements behave more like texture density discrimination, dues to effects similar to visual crowding (Anobile et al., 2015, 2016; Burr et al., 2017).

The results are very consistent with two prominent theories of autistic perception that emphasize differences in global versus local processing: the *enhanced perceptual functioning account* (Mottron et al., 2006), which posits enhanced and increased autonomy of perception promoting local processing, and the *weak central coherence theory*, which proposes that autistic individuals fail to extract overall meaning from visual displays, resulting in a reduced awareness of the global aspects of stimuli, combined with a relatively heightened awareness of the details or parts of stimuli (Happé & Frith, 2006). Both of these theories predict that autistic perception systems would be less prone to automatically group the connected dots into a single item, reducing overall numerosity. Enhanced perceptual functioning would tend to emphasize more the individual dots, and weak coherence would predict weaker propensity for active grouping strategies into meaningful segmented objects.

The results are also broadly consistent with Pellicano and Burr’s (2012) and others (Friston et al., 2013; Lawson et al., 2014; Palmer et al., 2017; van de Cruys et al., 2014) more recent theories that autistic perception relies more on primary sensory data than on contextual cues. The theory was couched largely in terms of Bayesian perceptual *priors*, knowledge of the world built up over various timescales. However, many forms of contextual information contribute to the construction of the percept, including the grouping cues of connected-dot stimuli, which help segment the scene into meaningful objects. If the reliance on contextual information is generally less in autism, and with people with strong autistic-like traits, then the magnitude of the connectiveness effect should vary inversely with AQ, as reported here.

Although the connected-dot patterns seem to activate at least partially different perceptual strategies in high and low-AQ participants, both had very similar precision in discrimination, suggesting that the difference in connectedness effects does not reflect inattention or some other more generic difficulties with judging numerical quantities. This result is in line with previous evidence showing that although adaptation to number, and also to face identities, was significantly reduced in autistic children, the discrimination precision was as good as for neurotypical children (Pellicano et al., 2007; Turi et al., 2015). Thus, while there are clearly autism-related differences in the strategies used for estimating and discriminating numerosities, these did not lead to measurable differences in discrimination precision, suggesting that both strategies are equally effective. It also suggests that the strategies of any individual remain relatively constant over time: if they were to vary, from strong to weak connectivity-induced bias, it would result in much broader psychometric functions, and hence high Weber fractions.

This study introduces to autism research a novel test of perceptual grouping, in which an objective measure of the strength of the grouping is strongly predicted by autistic-like traits. There are mainly ways to measure perceptual grouping, including classic Gestalt tests (Ben-Av & Sagi, 1995; Dresp, 1993; Han et al., 1999; Ogden, 1923). However, the advantage of the current technique is that it taps grouping mechanisms indirectly, by asking participants to report not on grouping or perceptual organization, but on apparent numerosity. That the measurement is indirect makes it less open to various cognitive biases or ambiguities in instructions, which can be problematic in estimating higher level perceptual attributes. Judgements of numerosity are very intuitive and spontaneous, as revealed by higher sensitivity compared with density or area judgments (Cicchini et al., 2016), and faster orienting saccades (Castaldi et al., 2020), rendering them ideal for clinical testing.

Another advantage of using the connected-dot paradigm is that it has been extensively studied in neurotypical adults, using both psychophysical and neurophysiological techniques. Connecting dots changes not only the apparent numerosity of a dot-cloud, but also fMRI adaptation to number (He et al., 2009), which follows the apparent, not the physical numerosity, as does psychophysical adaptation (Fornaciai et al., 2016). The connectedness effect can also be revealed by pupillometry (Castaldi et al., 2021), a non-invasive technique very suitable for clinical populations. All studies show that the connectedness bias does not result from response biases or cognitive artifacts but reflects real neuronal changes: and these neuronal changes covary strongly with autistic-like personality traits.

Measuring neurotypical participants with variable degrees of autistic-like traits is relatively simple and can be predictive of results with diagnosed autistic patients in line with the idea of a dimensional concept of autistic traits, distributed along a continuum across the whole population, of which the clinical sample forms an extreme (Bailey et al., 2013; Baron-Cohen et al., 2001; Chouinard et al., 2013; Constantino & Todd, 2003; Ruzich et al., 2015; Skuse et al., 2009; Wheelwright et al., 2010). However, the ultimate goal of this line of research is to understand better the autistic brain. The next step is obviously to test a clinical population diagnosed with autism, to see if the effect extends into the clinical population. This should be feasible, given the simplicity of the task, which can also be easily adapted to a child-friendly game to study autism in development. Numerosity has proven to be a useful tool to study autistic perception, as it taps mid-high level mechanisms with a simple and intuitive task, yielding acuity thresholds as low in autistic participants as in neurotypicals (Turi et al., 2015). That connectively also affects objective measures such as fMRI adaptation and pupillary response makes it probably that it taps genuine perceptual differences, rather than simple response biases.

In conclusion, this study on the effects of grouping on extracting high-order information from the visual field may add an important piece to the puzzle of how visual processing differs in autism and autistic-like personality traits. These general differences in how participants with variable degree of autistic traits may see the world—more or less susceptible to perceptual grouping—could underlie the representation of multiple elements in several ways, impacting how people with autism do important visual tasks, such as interpreting social scenes.

## References

[CR1] Anobile G, Cicchini GM, Burr DC (2016). Number as a primary perceptual attribute: A review. Perception.

[CR2] Anobile G, Cicchini GM, Pomè A, Burr DC (2017). Connecting visual objects reduces perceived numerosity and density for sparse but not dense patterns. Journal of Numerical Cognition.

[CR3] Anobile G, Turi M, Cicchini GM, Burr DC (2015). Mechanisms for perception of numerosity or texture-density are governed by crowding-like effects. Journal of Vision.

[CR4] Arnheim R, Metzger W (1977). Gesetze des Sehens. Leonardo.

[CR5] Bailey, A., Le Couteur, A., Gottesman, I., Bolton, P., Simonoff, E., Yuzda, E., & Rutter, M. (2013). Autism as a strongly genetic disorder: Evidence from a British twin study. *The science of mental health: Volume 2—autism.*Psychological medicine. 10.1017/s0033291700028099 7792363

[CR6] Baron-Cohen S, Wheelwright S, Skinner R, Martin J, Clubley E (2001). The autism-spectrum quotient (AQ): Evidence from asperger syndrome/high-functioning autism, males and females, scientists and mathematicians. Journal of Autism and Developmental Disorders.

[CR7] Ben-Av MB, Sagi D (1995). Perceptual grouping by similarity and proximity: Experimental results can be predicted by intensity autocorrelations. Vision Research.

[CR8] Brainard DH (1997). The psychophysics toolbox. Spatial Vision.

[CR9] Burr DC, Anobile G, Arrighi R (2017). Psychophysical evidence for the number sense. Philosophical Transactions of the Royal Society B.

[CR10] Castaldi E, Burr D, Turi M, Binda P (2020). Fast saccadic eye-movements in humans suggest that numerosity perception is automatic and direct: Numerosity drives fast saccades. Proceedings of the Royal Society B.

[CR11] Castaldi, E., Pomè, A., Cicchini, G. M., Burr, D. C., & Binda, P. (2021). Pupil size automatically encodes numerosity, Nature Communications (*under review*) 10.1038/s41467-021-26261-4 PMC8511033 34642335

[CR12] Chouinard PA, Noulty WA, Sperandio I, Landry O (2013). Global processing during the Müller-Lyer illusion is distinctively affected by the degree of autistic traits in the typical population. Experimental Brain Research.

[CR13] Cicchini GM, Anobile G, Burr DC (2016). Spontaneous perception of numerosity in humans. Nature Communications.

[CR14] Constantino JN, Todd RD (2003). Autistic traits in the general population: A twin study. Archives of General Psychiatry.

[CR15] Dresp B (1993). Bright lines and edges facilitate the detection of small light targets. Spatial Vision.

[CR16] Fornaciai M, Cicchini GM, Burr DC (2016). Adaptation to number operates on perceived rather than physical numerosity. Cognition.

[CR17] Franconeri SL, Bemis DK, Alvarez GA (2009). Number estimation relies on a set of segmented objects. Cognition.

[CR18] Friston KJ, Lawson R, Frith CD (2013). On hyperpriors and hypopriors: Comment on Pellicano and Burr. Trends in Cognitive Sciences.

[CR19] Han S, Humphreys GW, Chen L (1999). Uniform connectedness and classical gestalt principles of perceptual grouping. Perception and Psychophysics.

[CR20] Happé F, Frith U (2006). The weak coherence account: Detail-focused cognitive style in autism spectrum disorders. Journal of Autism and Developmental Disorders.

[CR21] He L, Zhang J, Zhou T, Chen L (2009). Connectedness affects dot numerosity judgment: Implications for configural processing. Psychonomic Bulletin & Review.

[CR22] He L, Zhou K, Zhou T, He S, Chen L (2015). Topology-defined units in numerosity perception. Proceedings of the National Academy of Sciences USA.

[CR23] Kirjakovski A, Matsumoto E (2016). Numerosity underestimation in sets with illusory contours. Vision Research.

[CR24] Lawson RP, Rees G, Friston KJ (2014). An aberrant precision account of autism. Frontiers in Human Neuroscience.

[CR25] Mottron L, Dawson M, Soulières I, Hubert B, Burack J (2006). Enhanced perceptual functioning in autism: An update, and eight principles of autistic perception. Journal of Autism and Developmental Disorders.

[CR26] Ogden RM (1923). Visuell wahrgenommene figuren. Studien in psychologischer analyse. Psychological Bulletin.

[CR27] O’Riordan M, Plaisted K (2001). Enhanced discrimination in autism. Quarterly Journal of Experimental Psychology Section A.

[CR28] Palmer CJ, Lawson RP, Hohwy J (2017). Bayesian approaches to autism: Towards volatility, action, and behavior. Psychological Bulletin.

[CR29] Pellicano E, Burr D (2012). When the world becomes “too real”: A Bayesian explanation of autistic perception. Trends in Cognitive Sciences.

[CR30] Pellicano E, Jeffery L, Burr D, Rhodes G (2007). Abnormal adaptive face-coding mechanisms in children with autism spectrum disorder. Current Biology.

[CR31] Plaisted K, Swettenham J, Rees L (1999). Children with autism show local precedence in a divided attention task and global precedence in a selective attention task. Journal of Child Psychology and Psychiatry and Allied Disciplines.

[CR32] Rouder JN, Speckman PL, Sun D, Morey RD, Iverson G (2009). Bayesian t tests for accepting and rejecting the null hypothesis. Psychonomic Bulletin and Review.

[CR33] Ruta L, Mazzone D, Mazzone L, Wheelwright S, Baron-Cohen S (2012). The autism-spectrum quotient—Italian version: A cross- cultural confirmation of the broader autism phenotype. Journal of Autism and Developmental Disorders.

[CR34] Ruzich E, Allison C, Smith P, Watson P, Auyeung B, Ring H, Baron-Cohen S (2015). Measuring autistic traits in the general population: A systematic review of the autism-spectrum quotient (AQ) in a nonclinical population sample of 6,900 typical adult males and females. Molecular Autism.

[CR35] Shah A, Frith U (1983). An islet of ability in autistic children: A research note. Journal of Child Psychology and Psychiatry.

[CR36] Shah A, Frith U (1993). Why do autistic individuals show superior performance on the block design task?. Journal of Child Psychology and Psychiatry.

[CR37] Skuse DH, Mandy W, Steer C, Miller LL, Goodman R, Lawrence K, Emond A, Golding J (2009). Social communication competence and functional adaptation in a general population of children: Preliminary evidence for sex-by-verbal IQ differential risk. Journal of the American Academy of Child and Adolescent Psychiatry.

[CR38] Turi M, Burr DC, Binda P (2018). Pupillometry reveals perceptual differences that are tightly linked to autistic traits in typical adults. eLife.

[CR39] Turi M, Burr DC, Igliozzi R, Aagten-Murphy D, Muratori F, Pellicano E (2015). Children with autism spectrum disorder show reduced adaptation to number. Proceedings of the National Academy of Sciences.

[CR40] van de Cruys S, Evers K, van der Hallen R, van Eylen L, Boets B, de-Wit L., & Wagemans J. (2014). Precise minds in uncertain worlds: Predictive coding in autism. Psychological Review.

[CR41] Wagemans J, Elder JH, Kubovy M, Palmer SE, Peterson MA, Singh M, von der Heydt R (2012). A century of Gestalt psychology in visual perception: I. Psychological Bulletin.

[CR42] Watson AB, Pelli DG (1983). QUEST: A Bayesian adaptive psychometric method. Perception & Psychophysics.

[CR43] Wertheimer, M. (1938). Laws of organization in perceptual forms. In W. D. Ellis (Ed.), *A source book of Gestalt psychology.*Kegan Paul, Trench, Trubner & Company.

[CR44] Wheelwright S, Auyeung B, Allison C, Baron-Cohen S (2010). Defining the broader, medium and narrow autism phenotype among parents using the autism spectrum quotient (AQ). Molecular Autism.

